# Expert and deep learning model identification of iEEG seizures and seizure onset times

**DOI:** 10.3389/fnins.2023.1156838

**Published:** 2023-07-05

**Authors:** Sharanya Arcot Desai, Muhammad Furqan Afzal, Wade Barry, Jonathan Kuo, Shawna Benard, Christopher Traner, Thomas Tcheng, Cairn Seale, Martha Morrell

**Affiliations:** ^1^NeuroPace, Inc., Mountain View, CA, United States; ^2^Department of Neurology, University of Southern California, Los Angeles, CA, United States; ^3^Department of Neurology, Yale University, New Haven, CT, United States; ^4^Department of Neurology and Neurological Sciences, Stanford University, Stanford, CA, United States

**Keywords:** seizure classification, big data, EEG, epilepsy, deep learning

## Abstract

Hundreds of 90-s iEEG records are typically captured from each NeuroPace RNS System patient between clinic visits. While these records provide invaluable information about the patient’s electrographic seizure and interictal activity patterns, manually classifying them into electrographic seizure/non-seizure activity, and manually identifying the seizure onset channels and times is an extremely time-consuming process. A convolutional neural network based Electrographic Seizure Classifier (ESC) model was developed in an earlier study. In this study, the classification model is tested against iEEG annotations provided by three expert reviewers board certified in epilepsy. The three experts individually annotated 3,874 iEEG channels from 36, 29, and 35 patients with leads in the mesiotemporal (MTL), neocortical (NEO), and MTL + NEO regions, respectively. The ESC model’s seizure/non-seizure classification scores agreed with the three reviewers at 88.7%, 89.6%, and 84.3% which was similar to how reviewers agreed with each other (92.9%–86.4%). On iEEG channels with all 3 experts in agreement (83.2%), the ESC model had an agreement score of 93.2%. Additionally, the ESC model’s certainty scores reflected combined reviewer certainty scores. When 0, 1, 2 and 3 (out of 3) reviewers annotated iEEG channels as electrographic seizures, the ESC model’s seizure certainty scores were in the range: [0.12–0.19], [0.32–0.42], [0.61–0.70], and [0.92–0.95] respectively. The ESC model was used as a starting-point model for training a second Seizure Onset Detection (SOD) model. For this task, seizure onset times were manually annotated on a relatively small number of iEEG channels (4,859 from 50 patients). Experiments showed that fine-tuning the ESC models with augmented data (30,768 iEEG channels) resulted in a better validation performance (on 20% of the manually annotated data) compared to training with only the original data (3.1s vs 4.4s median absolute error). Similarly, using the ESC model weights as the starting point for fine-tuning instead of other model weight initialization methods provided significant advantage in SOD model validation performance (3.1s vs 4.7s and 3.5s median absolute error). Finally, on iEEG channels where three expert annotations of seizure onset times were within 1.5 s, the SOD model’s seizure onset time prediction was within 1.7 s of expert annotation.

## 1. Introduction

Clinical management of epilepsy is fraught with challenges (Dalic and Cook, 2016). One of them is the difficulty with objectively assessing patient responses to treatments since patient self-reports of seizures are unreliable (Hoppe et al., 2007). Baseline ambulatory iEEG recordings captured using implanted devices such as the NeuroPace RNS System contain features that correlate with clinical outcomes (Skarpaas et al., 2018; Desai et al., 2019a) and can be used as an objective data source for supplementing patient self-reports of seizures. Although not necessarily indicative of a clinical seizure, another straightforward and objective measure is simply the numbers and types of electrographic seizures captured in the ambulatory iEEG recordings. Comparing numbers and types of electrographic seizures before and after treatment changes could potentially be a reliable indicator of the patient’s clinical response. Counts of electrographic seizures in ambulatory iEEG recordings can have diagnostic uses too. For example, if electrographic seizures are restricted to certain channels in the ambulatory multi-channel iEEG recordings, the patient may be identified as a candidate for resection surgery (King-Stephens et al., 2015). Thus, identifying electrographic seizures in iEEG recordings is valuable and of particular interest to physicians.

Hundreds of 90-s, 4-channel iEEG records are typically captured from each patient treated with the RNS System between clinic visits, which are generally 3–6 months apart. Manual review of these iEEG records prior to every visit adds significant time to the clinic workflow, and can lead to reviewer fatigue and increased errors in iEEG review. Additionally, manual iEEG review can be subjective in nature (Halford et al., 2015). Differences in EEG training and varying levels of experience may contribute to differences in manual iEEG interpretations. Automated methods for classifying iEEG recorded electrographic seizures and identifying channels with the earliest seizure onsets could (1) simplify iEEG review and speed up clinic workflows, (2) facilitate objective iEEG analysis, and (3) contribute to better clinical outcomes.

Electrographic seizure patterns can vary substantially from patient to patient (Haas et al., 2007). Manually determining rules to identify each type of electrographic seizure and seizure onset pattern is a daunting process, with machine learning models trained using hand-engineered features having limited practical applications. For example, machine learning models are frequently trained to classify iEEG data within a single patient or a small group of patients (Thodoroff et al., 2016; Acharya et al., 2018; Ansari et al., 2019; Roy et al., 2019). A generic model that can work out-of-the box for many patient and seizure types would have greater practical usability.

Deep learning models that can directly learn patterns from large amounts of data have made great strides in recent years (LeCun et al., 2015). For example, transformer-based models can take a text prompt and generate relevant synthetic videos automatically (Singer et al., 2022). Language models capable of understanding human conversations can reply to complex verbal queries (Brown et al., 2020). In the domain of computer vision, self-driving cars serve as a prime example of the progress made with deep convolutional neural networks (CNN) (Chen and Huang, 2017; Totakura et al., 2021). Even in the medical field, several studies have leveraged deep neural networks to identify abnormalities in MRIs, mammograms, and retinal images with a high degree of confidence (Ting et al., 2017; Lundervold and Lundervold, 2019; Yala et al., 2019). Several medical diagnostic tools with “AI in the loop” (AI, Artificial Intelligence) that aid physicians in making clinical decisions have also been recently approved by the FDA (Benjamens et al., 2020). Thus, the data science tools required for learning complex patterns directly from data are available and have demonstrated successful practical applications in several domains.

Big datasets are central to all these models. With the NeuroPace RNS System (Jarosiewicz and Morrell, 2021), over 10 million iEEG records have been captured from >4,500 patients. Out of this, ~1 million iEEG records have been captured from 256 patients enrolled in the RNS System feasibility, pivotal, and LTT clinical trials (Sun and Morrell, 2014; Nair et al., 2020). Large datasets offer the opportunity to train deep learning models directly on the dataset without the need for extracting hand-engineered features and creating hand-engineered rules. By manually labeling a fraction of the RNS System clinical trial dataset (~138,000 iEEG records from 113 patients), an electrographic seizure classifier model was trained with 95.7% class-balanced classification accuracy in test patients (iEEG records from 20% of 113 patients). The entire clinical trial dataset could not be manually labeled because of resource constraints. A flowchart showing RNS System iEEG data split into training and test datasets is available in a previous publication by our group (Barry et al., 2021). Since model training and testing was performed on the dataset annotated by the same individual, it is not known how the model would fair against reviewer physicians’ annotations in a practical setting. Further, it is not known how the model’s agreement with reviewers would compare with reviewer agreement with other reviewers. Finally, it is not known if the trained models would have similar performance in different patient populations, and on data captured from a newer version of the neurostimulator. To address these questions, a new test dataset of iEEG records was created with annotations from three independent expert reviewers. Reviewer agreement with each other as well as model agreement with the reviewers are reported.

In addition to identifying electrographic seizures in iEEG records, physicians often also manually identify seizure onset times. This information is valuable for studying seizure spread patterns and for configuring neurostimulator detection settings for responsive closed-loop stimulation. The task of manually identifying seizure onset times is particularly time-consuming since brain activity patterns at the onset of seizures can evolve slowly and often requires reviewing the activity at multiple time and frequency scales (Spencer et al., 1992; Lee et al., 2000; Perucca et al., 2014; Nune et al., 2019). Hence, an automated method for identifying seizure onset times is desirable. Training a reliable deep learning-based seizure onset time detection model from scratch would require a large number of manually annotated examples of seizure onset times from many patients. Alternatively, transfer-learning techniques could be applied to fine-tune a previously trained model with a relatively small, annotated training dataset (Weiss et al., 2016). Since the electrographic seizure classifier model was trained on a large multi-patient dataset, it could serve as an excellent starting point for solving the problem of identifying seizure onset times. In this paper, we explore this idea with data augmentation techniques applied to a relatively small, annotated dataset for seizure onset time estimation.

This paper significantly adds to existing literature on leveraging deep learning and machine learning techniques for facilitating data review in epilepsy. First, it quantifies expert seizure classification agreements in patients with seizure onsets in mesial temporal and in neocortical regions. Second, it compares the trained electrographic seizure classifier scores against each reviewer individually and against all three reviewers grouped together. Third, it demonstrates that the seizure classifier model can serve as an excellent starting-point model for solving other related problems with small, labeled datasets.

## 2. Methods

### 2.1. The RNS system and iEEG records

**Figure 1:**
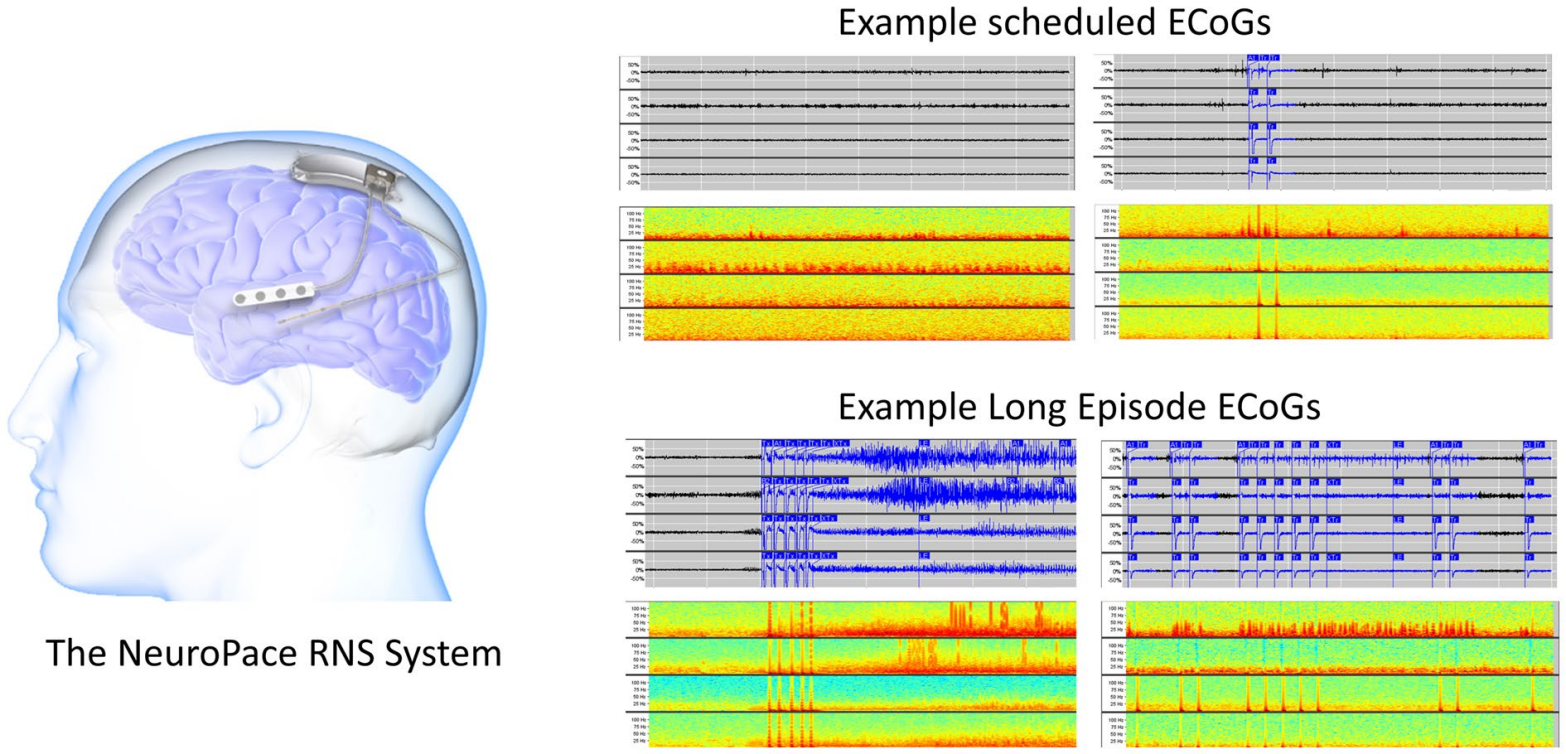
The RNS System (left). Two example 90-s scheduled iEEG records in time-series and spectrogram representation (right top). Two example 90-s long episode (LE) iEEG records in time-series and spectrogram representation (right bottom).

The NeuroPace RNS System (Figure 1) is FDA approved for the treatment of drug-resistant partial onset epilepsy in adults 18 years and older (Jarosiewicz and Morrell, 2021). Details about the RNS System have been published in numerous previous studies (Skarpaas et al., 2018; Desai et al., 2019a,b, 2022; Barry et al., 2021). Briefly, the device delivers responsive neurostimulation when patient-specific abnormal brain patterns are detected. Programming the responsive neurostimulator with patient-specific detection patterns is performed by the patient’s physician. Physicians also program the stimulation settings, which include the stimulation frequency, charge density, burst duration, pulse width, and stimulation pathways.

In addition to stimulating abnormal activity, the device also captures short iEEG recordings of brain activity. Each iEEG record contains 4-channels of iEEG data with each channel sampled at 250 Hz. The two major types of iEEG records captured by the device are long episode (LE) iEEG records and scheduled iEEG records (Figure 1). As the name suggests, storage of LE iEEG records is triggered when long durations of abnormal activity are detected by the device. Many, but not all, of the LE iEEG records contain electrographic seizures. Scheduled iEEG records, on the other hand, are captured at scheduled times every day, typically at 2 a.m. and 2 p.m., and usually contain baseline activity but rarely electrographic seizures.

### 2.2. The electrographic seizure classifier model

Each channel of activity in 138,000 iEEG records was manually annotated as electrographic seizure or non-seizure by a NeuroPace employee (Barry et al., 2021). An iEEG record clustering tool was used to speed up the process of labeling such a large dataset. Three other employees, including the Chief Medical Officer of NeuroPace, an MD who is board certified in epilepsy, were consulted when the activity could not be annotated with a high degree of confidence. iEEG channels from 80% of 113 patients were used for training a series of convolutional neural network (CNN) models to perform the binary classification task of classifying each iEEG channel as electrographic seizure or not. The best model, a ResNet50 architecture-based model, had a class-balanced classification accuracy of 95.7%, and F_1_ score of 94.3% on iEEG records from the test dataset (iEEG records from the remaining 20% of patients). Details about the unsupervised iEEG clustering process, time taken to manually label each channel in 138,000 iEEG records, and methods used for training the CNN models can be found in a previous publication (Barry et al., 2021).

### 2.3. Sample size computations to determine the number of iEEGs records needed in the clinical validation dataset

A statistician was consulted to compute the sample size required to clinically validate the trained electrographic seizure classifier. Sample size computations were performed assuming a true model classification rate of at least 85%, target performance goal of at least 70%, and within patient correlation of classification scores in the range of 0.4–0.7. Selection of these parameters was guided by *ad hoc* analyses performed on the RNS System dataset, and a previous study (Onorati et al., 2021), which aimed to clinically validate an electrographic seizure classifier model on continuous heart rate and electrodermal activity captured using a wrist worn device.

Anecdotally, it is known that between 30% and 40% of long episodes (LE) are electrographic seizures, while less than 5% of scheduled iEEG records contain electrographic seizures. Hence, to increase the chances of picking iEEG records with electrographic seizures when randomly selecting test iEEG records, selections were only made from the LE iEEG records from each patient. Additionally, since LE type iEEG records are the primary iEEG type that needs to be classified as an electrographic seizure or not in a practical setting, selecting only LE iEEG records for testing the performance of the trained models is appropriate. Subsequently, 10 LE iEEG records were randomly selected from each RNS System patient, and an assumption of including 2–6 electrographic seizures per patient was made for sample size calculations.

Given a target number of iEEG records required to perform the testing, selecting the iEEG records from a larger number of patients provided higher statistical power than selecting the same target number from fewer patients, which requires a larger number of iEEG records per patient (Table 1). Based on power calculations, a total of 1,000 iEEG records were randomly selected from 100 RNS System patients, with 10 randomly selected LE iEEG records from each patient.

**Table 1:** Sample size computations for a few scenarios with different numbers of assessments per patient, and different numbers of patients.

	Scenario 1	Scenario 2	Scenario 3	Scenario 4
Assumed true rate	85%	85%	85%	85%
Target performance goal (TPG)	70%	70%	70%	70%
Assumed within patient correlation	0.70	0.70	0.70	0.70
Assessments per patient (i.e., number of electrographic seizure iEEG records per patient)	6	4	2	3
Sample size (i.e., number of unique patients)	50	75	100	100
Power	79%	92%	94%	95.7%

### 2.4. Expert review of iEEG data

Three expert reviewers independently annotated each channel of brain activity in the 1,000 randomly chosen LE iEEG records from 100 RNS System patients. The 1,000 iEEG records were presented to the physicians in a randomized order *via* an iEEG record annotation web-based tool. A screenshot of the reviewer annotation tool is shown in supplementary material (Supplementary Figure S1). Time-series waveforms of the 4 channels of brain activity along with their spectrogram representation were presented. The iEEG annotation tool allowed physicians to zoom into the time-series waveforms or spectrograms at 5 different timescales—5 s, 10 s, 30 s, 60 s or full (i.e., to view the entire length of the iEEG record). Additionally, physicians could also vertically zoom in on the timeseries waveforms and spectrogram images.

The reviewers were asked to annotate each channel of each iEEG record as electrographic seizure, non-seizure, or unsure. On the electrographic seizure channels, the reviewers were additionally asked to annotate the seizure onset time at a resolution of 1/10th of a second or 1 decimal point.

Qualifications of the three reviewers recruited for labeling the test dataset are presented below.

Reviewer 1: Assistant Professor of Neurology, Epilepsy Division. Associate Fellowship Director, Clinical Neurophysiology/Epilepsy, Department of Neurology, Yale School of Medicine. Board certifications: Neurology, Clinical Neurophysiology, Epilepsy.

Reviewer 2: Assistant Professor of Neurology, Epilepsy Division, Department of Neurology, Keck School of Medicine. Board certifications: Neurology, Clinical Neurophysiology, Epilepsy.

Reviewer 3: Assistant Professor of Neurology, Epilepsy Division, Department of Neurology, Keck School of Medicine. Board certifications: Neurology, Epilepsy.

### 2.5. Test data set split

Out of the 100 patients included in the test dataset, 50 had the newer RNS-320 model of the RNS Neurostimulator implanted, while the remaining 50 patients had the older RNS-300 M model implanted. A deliberate decision was made to evenly split the test data between the two models of the RNS System to determine if the neurostimulator version influenced the trained model’s performance. Since the Electrographic Seizure Classifier (ESC) model (Barry et al., 2021) was trained entirely on data captured from the older RNS-300 M model, a lower performance on data captured from the RNS-320 might suggest data drift and the need for re-training the ESC model on data captured from the RNS-320.

Additionally, out of the 100 patients included in the test dataset, 36 had both RNS leads implanted in the mesiotemporal region, 29 had both leads implanted in the neocortical region, and the remaining 35 had leads implanted in both regions. The data split by lead location was not pre-determined. Instead, data were selected from 100 randomly picked patients to generally reflect the RNS System’s overall patient population.

### 2.6. Model vs. reviewer scores

#### 2.6.1. ESC model vs. reviewer scores on iEEG channels

The electrographic seizure classifier (ESC) model was re-trained on the same training datasets as before (Barry et al., 2021), using the same model architecture (ResNet50), but using an updated version of TensorFlow (version:2.6.0). The same five cross-validation data splits were performed to produce five versions of the electrographic seizure classifier models. Each iEEG channel in the reviewer-annotated iEEG records was converted to a spectrogram image by using the function matplotlib.pyplot.specgram with window size 256 and step size 128. The resulting spectrogram images were tested on all five versions of the trained ESC models. The trained models returned prediction scores in the range of 0–1. An operating point of 0.5 was selected for determining the ESC model’s prediction class of each individual iEEG channel. Results are also shown for an operating point of 0.8 for comparison purposes.

The methodology described in Scheuer et al. (2017, 2021) was followed to calculate pairwise sensitivity and false positive rates for inter-reviewer and reviewer-model pairs. In the inter-reviewer comparisons, each reviewer was used as a reference against which the other two reviewers were tested. Similarly, in the model to reviewer comparisons, each of the three reviewers was used as a reference, and the models were tested against them.

To evaluate whether the models were noninferior to the human reviewers (discussed in Scheuer et al., 2017), an accelerated bootstrap analysis (BCa) of the pairwise sensitivity and false positive rates for all patients was conducted. This approach resembled the one outlined in Scheuer et al. (2017). The 95% confidence intervals for the mean sensitivity and false positive rates were computed for each comparison (inter-reviewer and model to reviewer).

#### 2.6.2. ESC model vs. reviewer scores on iEEG records

In some applications, it may be sufficient to classify each iEEG record as seizure or not, without the need for knowing which iEEG channels contain the electrographic seizure. For comparing the model scores with reviewer scores at the iEEG record-level, the maximum seizure probability among the 1–4 (usually 4) iEEG channels was taken, and if it exceeded the model operating point, the iEEG record was classified as an electrographic seizure. Similarly, if the reviewers annotated any one of the channels in the iEEG record as an electrographic seizure, the iEEG record was considered to be an electrographic seizure record. Higher model operating points of 0.8 and 0.9 were selected for this task since the probability of false positives is high when all channels need to be classified as non-seizures for the multi-channel iEEG record to be classified as non-seizure, but only one channel needs to be classified as seizure for the iEEG record to be a classified as seizure.

#### 2.6.3. Seizure onset detection model training and validation

**Figure 2:**
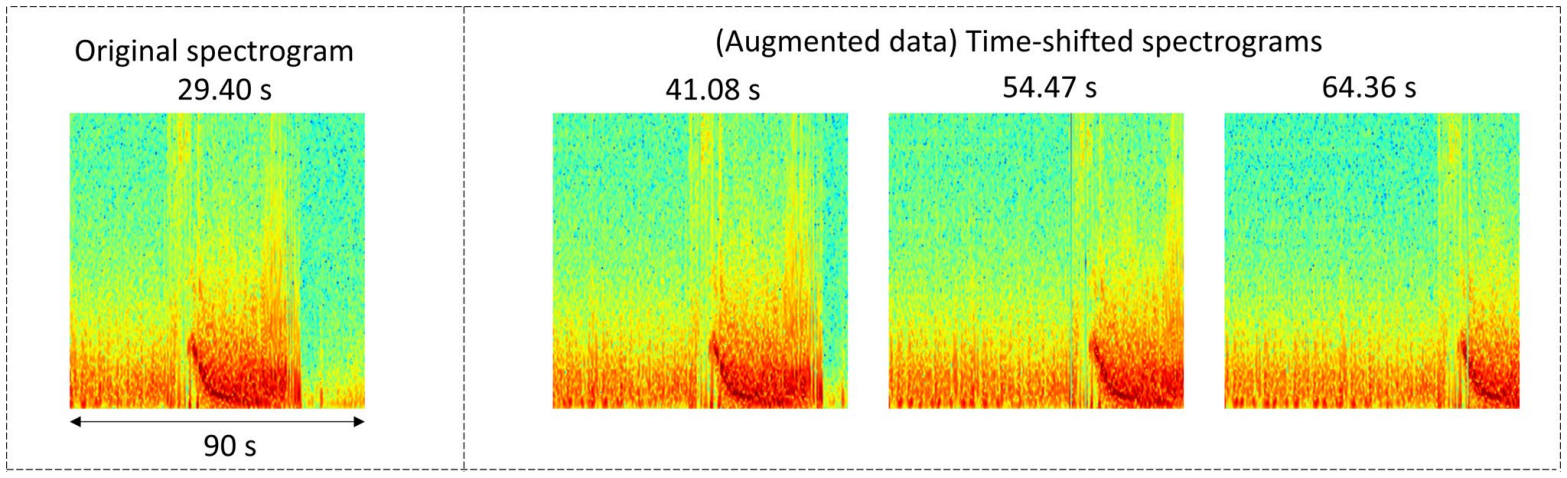
Example original and time-shifted (augmented) annotated iEEG channels used for training a seizure onset time detection model. The seizure onset time annotated in the original dataset was at 29.40 s. After time-shifting, the onset times in the augmented spectrograms were at 41.08, 54.47, and 64.36 s, respectively.

Seizure onset times were annotated on 4,859 iEEG channels from 50 RNS System patients by a NeuroPace employee. The annotated dataset was augmented by shifting the time series data points to create time-shifted versions of the same iEEG channel. Time-shifting was done several times on each iEEG channel but only in the one direction, with the electrographic activity shifted to the right by deleting several seconds of activity towards the end of the file and duplicating an equal amount of activity in the beginning of the file. The amount of right shift was randomly determined in ranges incrementing by 5 s. An example original spectrogram and a few time-shifted versions of the same spectrogram are shown in Figure 2. The data augmentation step produced a total of 30,768 spectrogram images.

Seizure activity was not time-shifted in the opposite direction (to the left) because in that case the resulting augmented images could look unrealistic if the seizure activity in the original image ended before the end of the file, since duplicating activity towards the end of the file could make the resulting image appear to contain 2 back-to-back seizure events, which could confuse model training.

The SOD model was trained with and without the augmented data to assess whether the data augmentation process contributed to increased model performance. Additionally, the ESC model’s weights were used as the starting point for fine-tuning, but also random weight initialization was tested, along with ImageNet weights (Deng et al., 2009) to check if the ESC weights offered any training advantage.

All layers of the ESC model were fine-tuned to make the SOD model. The code for fine-tuning the ESC model is provided in the Supplementary section. To train the SOD model, the final sigmoid activation layer of the ESC model was replaced with a dense layer which is appropriate for regression tasks. Adam optimizer was used with learning rate of 0.0001. Model training and validation experiments were performed on a Linux Ubuntu 18.04 machine using Tensorflow version 2.6.0. Following preliminary exploration of the hyperparameter space, model training was performed for 400 epochs in each training fold. Five-fold cross-validation was performed with 80% of the data used for training and 20% used for testing. Models were saved after every epoch of training, and the saved model which had the lowest median absolute error on the held-out validation dataset was selected in each cross-validation fold. The selected SOD models were tested on iEEG channels with reviewer-annotated seizure onset times.

## 3. Results

### 3.1. Reviewer labeling of 1,000 iEEG records as electrographic seizure or non-seizure

Reviewers 1, 2, and 3 annotated 98.2%, 99.7%, and 98.9%, respectively, of the 1,000 randomly selected LE iEEG records as electrographic seizure or non-seizure. A small number of iEEG records were not annotated by the reviewers primarily because the reviewer was not able to decide what the activity represented. Overall, iEEG annotations from all three reviewers were available on 978 or 97.8% of the iEEG records. The numbers of iEEG records and channels annotated by the three reviewers is shown in Table 2.

**Table 2:** Numbers of iEEG records and channels annotated as electrographic seizure or non-seizure by the three reviewers.

	Reviewer 1	Reviewer 2	Reviewer 3
Number of iEEG records annotated	982	997	989
Number of iEEG channels annotated	3,927	3,976	3,923
Number of iEEG records annotated by all three reviewers	978		
Number of iEEG channels annotated by all three reviewers	3,874		

### 3.2. Reviewer agreement with each other, and reviewer agreement with the trained ESC model

#### 3.2.1. iEEG channels where all three reviewers agreed

**Figure 3:**
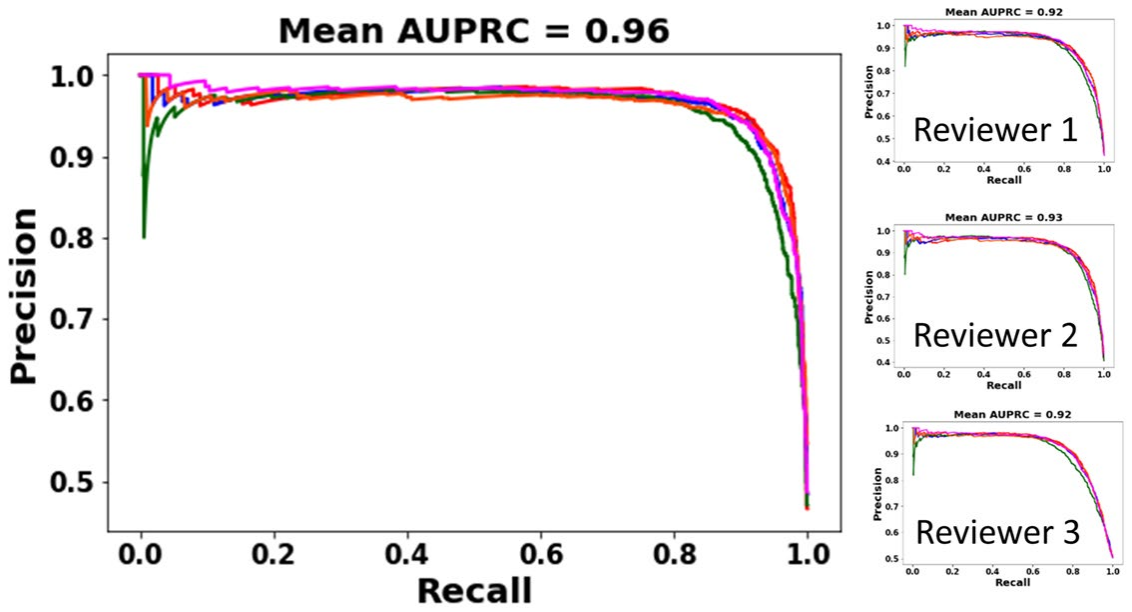
PR (Precision-Recall) curves of the 5 ESC models (each model trained on data from one cross-validation split) on a subset of iEEG channels in which all three reviewers’ annotations matched (left). PR curves and AUPRC of the ESC models against each individual reviewer (right).

A subset of iEEG channels on which all three reviewers provided the same (electrographic seizure or non-seizure) annotation (3,225 or 83.24% of iEEG channels) was selected. These represent iEEG channels with clear seizure or non-seizure activity. The classification performance of the trained ESC model on this subset of iEEG channels was analyzed using precision-recall curves and class-balanced accuracy metrics. All 5 versions of the trained ESC models (i.e., models from the 5 cross-validation folds) were applied to the test dataset resulting in 5 precision-recall curves as shown in Figure 3. The mean AUPRC (Area Under the Precision Recall Curve) of the 5 precision-recall curves was 0.96. In comparison, the AUPRC of a model with chance level performance would be 0.50.

On the subset of iEEG channels classified by all three experts as electrographic seizure or non-seizure (*n* = 3,225), the trained ESC models had an overall agreement of 92.5% at a model operating point of 0.5 (Table 3A), and an overall agreement of 93.19% at a model operating point of 0.8 (Table 3B). As expected, higher agreement on seizure channels only (95.2%) was observed with a lower operating point (0.5) since the model is more easily able to classify iEEG channels as electrographic seizures with a lower seizure threshold, and a higher agreement on non-seizure channels only was observed (95.4%) with a higher operating point (0.8). Model agreement with experts on iEEG channels split by lead location is also shown in Tables 3A,B at the two operating points of 0.5 and 0.8, respectively. At both operating points, the trained ESC models had significantly better classification performance (*p* < 0.05, independent *t*-test) on MTL leads compared to Neocortical and MTL + Neocortical leads. Agreement between the ESC model and experts in each training fold for each lead location is shown in the Supplementary section. The trained models had comparable performance on both models of RNS neurostimulations (model 300 M and 320) (Table 3C).

**Table 3:** Agreement between ESC model and reviewers on iEEG channels with full reviewer agreement.

A. Operating point = 0.5, split by lead location			
Lead location	Seizure agreement	Non-seizure agreement	Overall agreement (%)
All	95.2% (*n* = 1,417)	89.83% (*n* = 1,808)	92.52
MTL	97.06% (*n* = 626)	93.91% (*n* = 584)	95.48
NEO	91.36% (*n* = 338)	84.76% (*n* = 538)	88.46
MTL + NEO	94.50% (*n* = 453)	90.35% (*n* = 686)	92.92

B. Operating point = 0.8, split by lead location			
Lead location	Seizure agreement	Non-seizure agreement	Overall agreement (%)
All	90.98% (*n* = 1,417)	95.40% (*n* = 1,808)	93.19
MTL	94.73% (*n* = 626)	97.64% (*n* = 584)	96.18
NEO	84.85% (*n* = 338)	92.23% (*n* = 538)	88.54
MTL + NEO	90.38% (*n* = 453)	95.98% (*n* = 686)	93.18

C. Operating point = 0.5, split by RNS neurostimulator model			
Model	Seizure agreement	Non-seizure agreement	Overall agreement (%)
All	95.2% (*n* = 1,417)	89.83% (*n* = 1,808)	92.52
RNS-300 M	93.63% (*n* = 800)	90.23% (*n* = 850)	91.93
RNS-320	97.25% (*n* = 617)	89.48% (*n* = 958)	93.36

Data is split by lead location, and RNS neurostimulator model.

#### 3.2.2. Agreement on all iEEG channels

**Figure 4:**
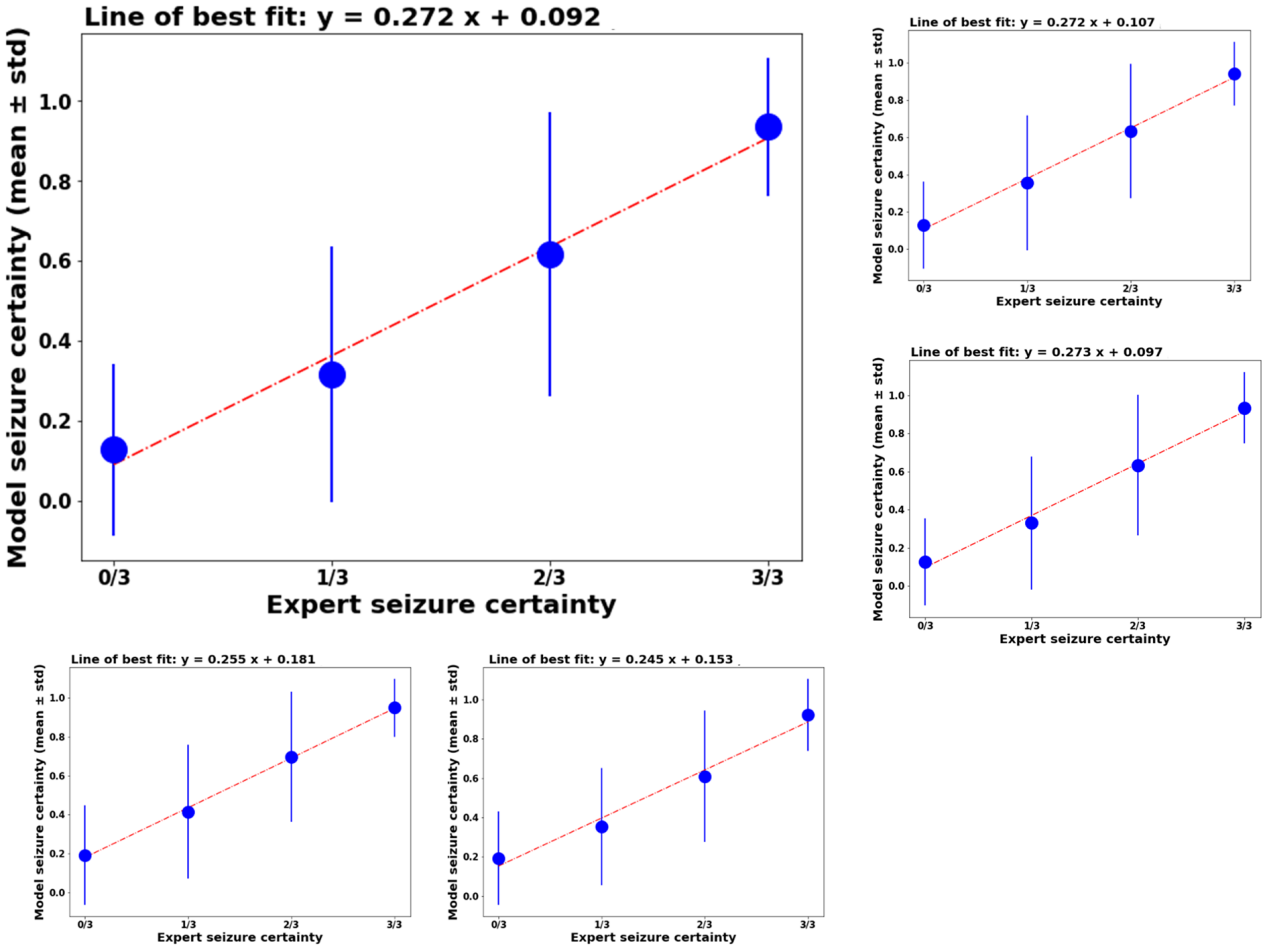
Left top panel: model certainty of the ESC model trained on fold 1 of the training data (y-axis) vs combined reviewer certainty (x-axis). Other panels: similar performance on the other 4 folds of the model training.

Model classification certainty was compared against combined reviewer classification certainty. Out of 3,874 iEEG channels annotated by all three reviewers, 1,808 or 46.67% of iEEG channels were annotated as non-seizure by all three reviewers. In other words, the combined reviewer-provided seizure probability on 46.67% of all annotated iEEG channels was 0. The trained ESC models gave these iEEG channels a mean seizure probability score in the range [0.12–0.19]. Figure 4 shows mean ± std. model probability scores vs. combined reviewer probability scores. Seizure probability scores from the 5 splits of ESC model are shown in the Supplementary section. In 382 or 9.8% of the iEEG channels, only 1 out of three reviewers classified the iEEG channels as showing an electrographic seizure. On these channels, the overall reviewer-provided seizure probability was 1/3 or 0.33, and ESC model’s means seizure probability score was in the same range [0.32–0.42]. Similarly, 215 or 5.5% IEEG channels were classified as electrographic seizures by 2 out of the three reviewers, giving these iEEG channels a combined reviewer-provided seizure probability value of 2/3 or 0.66. On these channels, the 5 versions of the ESC models produced mean seizure probability scores in the range [0.61–0.70]. Finally, the remaining 1,417 or 36.6% of IEEG channels were classified by all three reviewers as electrographic seizure, giving them a combined reviewer-provided seizure probability score of 1.0. The 5 versions of the ESC models gave these iEEG channels mean seizure probability in the range [0.92–0.95].

The ESC model’s seizure probability scores had a linear relationship with reviewer probability scores. A linear line of best fit was calculated for ESC model scores vs. reviewer model scores and are shown above each plot in Figure 4. In all cases, the *r*^2^ values (a measure of goodness of fit) was ≥0.98.

The ESC model’s iEEG channels classification agreement with each individual reviewer was at the same level as the reviewer’s agreement with other reviewers (Table 4). Reviewer 1 annotated a total of 3,927 iEEG channels. Out of these, 41.7% were annotated as electrographic seizures while the remaining 58.3% were annotated as non-electrographic seizures. Reviewer 1’s overall agreement with reviewers 2 and 3 was 92.9% and 86.9%, respectively. Against the ESC model, the average (of 5 ESC model versions) overall agreement was at 86.8% with the seizure and non-seizure annotation agreements at 90.4% and 84%, respectively. Reviewer 1’s agreements with the other 2 reviewers and the ESC models were higher on the MTL channels compared to neocortical channels.

**Table 4:** iEEG channel classification agreement between reviewers and ESC model.

	**Agreement with**			
**Reviewer 1**	**Reviewer 2**	**Reviewer 3**	**ESC model (0.5)**	**ESC model (0.8)**
All channels (3,927) (sz: 1,638, non-sz: 2,289)	92.94% (89.13, 95.67%)	86.91% (94.99, 81.12%)	86.81% (90.37, 84.26%)	88.68% (84.52, 91.67%)
MTL channels (1,416) (sz: 686, non-sz: 730)	95.48% (94.02, 96.85%)	88.49% (96.21, 81.23%)	91.36% (93.96, 88.91%)	92.25% (90.44, 93.97%)
NEO channels (1,151) (sz: 434, non-sz:717)	88.27% (80.86, 92.75%)	82.71% (91.47, 77.40%)	79.88% (84.05, 77.37%)	83.04% (75.55, 87.80%)
**Reviewer 2**	**Reviewer 1**	**Reviewer 3**	**ESC model (0.5)**	**ESC model (0.8)**
All channels (3,976) (sz: 1,571, non-sz: 2,405)	91.80% (92.93, 91.06%)	86.39% (96.69, 79.66%)	86.97% (92.40, 83.40%)	89.59% (87.20, 91.17%)
MTL channels (1,452) (sz: 675, non-sz: 777)	93.11% (95.55, 90.55%)	87.67% (97.19, 79.41%)	91.47% (95.20, 88.19%)	93.16% (92.40, 93.83%)
NEO channels (1,160) (sz: 405, non-sz: 755)	87.59% (86.67, 88.08%)	82.85% (94.57, 76.56%)	80.19% (87.07,76.52%)	84.31% (78.74, 87.28%)
**Reviewer 3**	**Reviewer 1**	**Reviewer 2**	**ESC model (0.5)**	**ESC model (0.8)**
All channels (3,923) (sz: 1,965, non-sz: 1,958)	86.99% (79.18, 94.84%)	87.56% (77.30, 97.85%)	85.04% (81.80, 88.33%)	84.30% (74.32, 94.38%)
MTL channels (1,421) (sz: 790, non-sz: 631)	88.18% (83.54, 93.98%)	89.58% (83.04, 97.78%)	88.79% (85.92, 92.40%)	88.0% (81.32, 96.41%)
NEO channels (1,144) (sz: 549, non-sz: 595)	83.22% (72.31, 93.27%)	84.0% (69.76, 97.14%)	80.31% (77.21, 83.12%)	79.15% (65.55, 91.54%)

Reviewer 2 annotated a total of 3,976 iEEG channels, with 39.5% (1,571) of the iEEG channels annotated as seizures and the remaining 60.5% (2,405) annotated as non-seizures. Out of the three reviewers, reviewer 2 annotated the smallest percentage of iEEG channels as seizures. Reviewer 2’s overall agreements with reviewers 1 and 3 was at 91.8% and 86.4%, with relatively high seizure agreements of 92.9% and 96.7%, respectively. Reviewer 2’s overall agreement with the ESC model was 86.8%, with seizure and non-seizure class agreements at 90.4% and 84.3%, respectively. Reviewer 2’s agreement with other reviewers and the ESC model was higher on MTL channels compared to neocortical channels.

Reviewer 3 annotated a total of 3,923 iEEG channels. A relatively high percentage (50.1%) of the total annotated iEEG channels were given the seizure class label by reviewer 3, with the remaining 49.9% annotated as non-seizures. Reviewer 3’s seizure class agreement with the other 2 reviewers was relatively low at 79.1% and 77.3%, respectively. Against the ESC model, reviewer 3’s overall, seizure, and non-seizure class agreements were at 85%, 81.8%, and 88.3%, respectively. Like reviewers 1 and 2, reviewer 3’s agreements with the other 2 reviewers were higher on MTL channels compared to neocortical channels. Overall, MTL channels on average had 5.65% greater agreement compared to neocortical channels in all three reviewers’ annotations (*p* < 0.05, paired *t*-test).

The ESC model’s agreement with each of the reviewers at a higher operating point of 0.8 is also shown in the table for comparison. In this case, model prediction probabilities ≥0.8 were assigned seizure class, and probabilities <0.8 were given the non-seizure class. As expected, the higher model operating point produced higher non-seizure agreements and lower seizure class agreements with all three reviewers.

Performance of the ESC model against each of the three reviewers is also shown using individual reviewer precision-recall curves (Figure 3 right panel). The AUPRC for Reviewers 1, 2, 3 are 0.92, 0.93, and 0.92, respectively.

**Figure 5:**
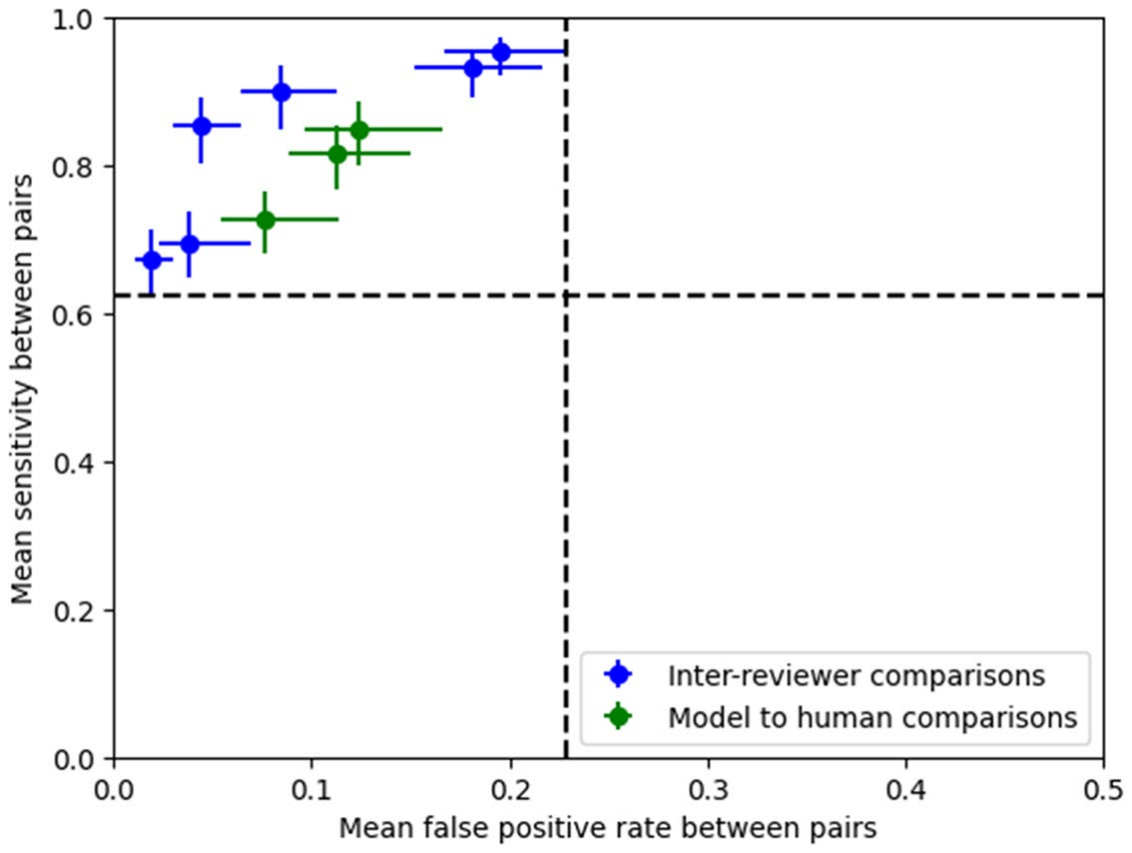
Pairwise sensitivity and false positive rate comparisons show that the model’s performance (operating point shown here is 0.8) lies within the 95% confidence intervals (dotted lines) of the expert pairs, thus demonstrating that the ESC model’s performance is non-inferior compared to the experts according to the methods outlined in Scheuer et al. (2017).

As described in Section 2, pairwise sensitivity and false positive rates were computed following methods described in Scheuer et al. (2017, 2021). Figure 5 illustrates that models demonstrated noninferiority compared to the human reviewers according to the criteria outlined in Scheuer et al. (2017).

#### 3.2.3. Classification agreement on iEEG records

Agreement between the ESC model and reviewers was studied at the iEEG record level (Table 5). As described in Section 2, if the reviewer annotated any of the channels in the iEEG record as seizure, the record was assigned the electrographic seizure annotation. Similarly, if the model predicted any of the channels as an electrographic seizure (at the selected operating points shown in Table 5), the model’s classification of the iEEG record would be to the electrographic seizure class. Otherwise, it would be to the non-seizure class. An operating point of 0.8 was selected for iEEG record-level classification, as computing the iEEG record level scores by combining individual channel scores would lead to high false positive rates since only one channel needs to be classified as a seizure for the entire multi-channel iEEG record to be classified as a seizure. The table also shows ESC record level classifications for an operating point of 0.9. Supplementary Figure S2 shows the effects of changing the operating point on the iEEG record-level classification agreements between each reviewer and the trained ESC model. As expected, at lower operating points, seizure class agreements are low and non-seizure class agreements are high. The overall agreements, however, are within a relatively narrow range (85%–90%).

**Table 5:** iEEG record classification agreement between reviewers and ESC model at 2 operating points (0.8 and 0.9).

	**Agreement with**			
**Reviewer 1**	**Reviewer 2**	**Reviewer 3**	**ESC model (0.8)**	**ESC model (0.9)**
Overall (982) (sz: 569, non-sz: 413)	91.85% (89.98, 94.43%)	87.52% (95.22, 77.00%)	88.17% (90.19, 85.38)	88.13% (87.21, 89.39)
**Reviewer 2**	**Reviewer 1**	**Reviewer 3**	**ESC model (0.8)**	**ESC model (0.9)**
Overall (997) (sz: 541, non-sz: 454)	91.85% (95.70, 87.24%)	87.65% (97.77, 75.50%)	89.4% (93.79, 84.12)	89.88% (91.05, 88.47)
**Reviewer 3**	**Reviewer 1**	**Reviewer 2**	**ESC model (0.8)**	**ESC model (0.9)**
Overall (989) (sz: 635, non-sz: 345)	87.79% (85.39, 92.17)	87.82% (82.99, 96.58)	85.62% (84.31, 87.98)	84.67% (80.88, 91.51)

### 3.3. Seizure onset detection model training and validation

#### 3.3.1. Comparing seizure onset prediction performance with augmented data vs. original dataset used for training

As described in Section 2, 4,859 iEEG channels annotated with seizure onset times were augmented by time-shifting the time-series waveforms and duplicating waveforms at the start of the iEEG channels. One of the 5 trained ESC models (the trained ESC model from 1st cross validation fold was randomly chosen) was fine-tuned using the augmented and original datasets and 5-fold cross-validation. The median absolute error on the validation dataset (i.e., iEEG channels from 20% of the patients not used for training in each of the 5 cross-validation folds) is shown in Figure 5 (top). The lowest median absolute error on the validation dataset for each of the 5 folds is shown in Table 6. Across the 5 training folds, the average median absolute error with the augmented data was significantly better (*p* < 0.05, paired *t*-test) when compared to training with only the original dataset. Overall, training with the augmented dataset led to an average median absolute error of 3.12 s compared to 4.4 s with the original dataset. Data augmentation improved the model’s prediction accuracy by 1.3 s.

**Table 6:** Median absolute error of the trained seizure onset detection model in each of the five training folds with and without augmented data used for training, and by training using different types of model weights as starting point.

Training fold number	Median absolute error				
	With data augmentation (and ESC weight initialization)	Without data augmentation (and ESC weight initialization)	ESC weight initialization (and data augmentation)	Random weight initialization (and data augmentation)	ImageNet weight initialization (and data augmentation)
1	3.51	4.62	3.51	5.19	3.96
2	3.05	4.33	3.05	4.44	3.39
3	2.47	3.86	2.47	4.95	2.72
4	3.38	5.05	3.38	4.20	3.69
5	3.38	4.13	3.38	4.83	3.69
Mean	3.12	4.40	3.12	4.72	3.49

**Figure 6:**
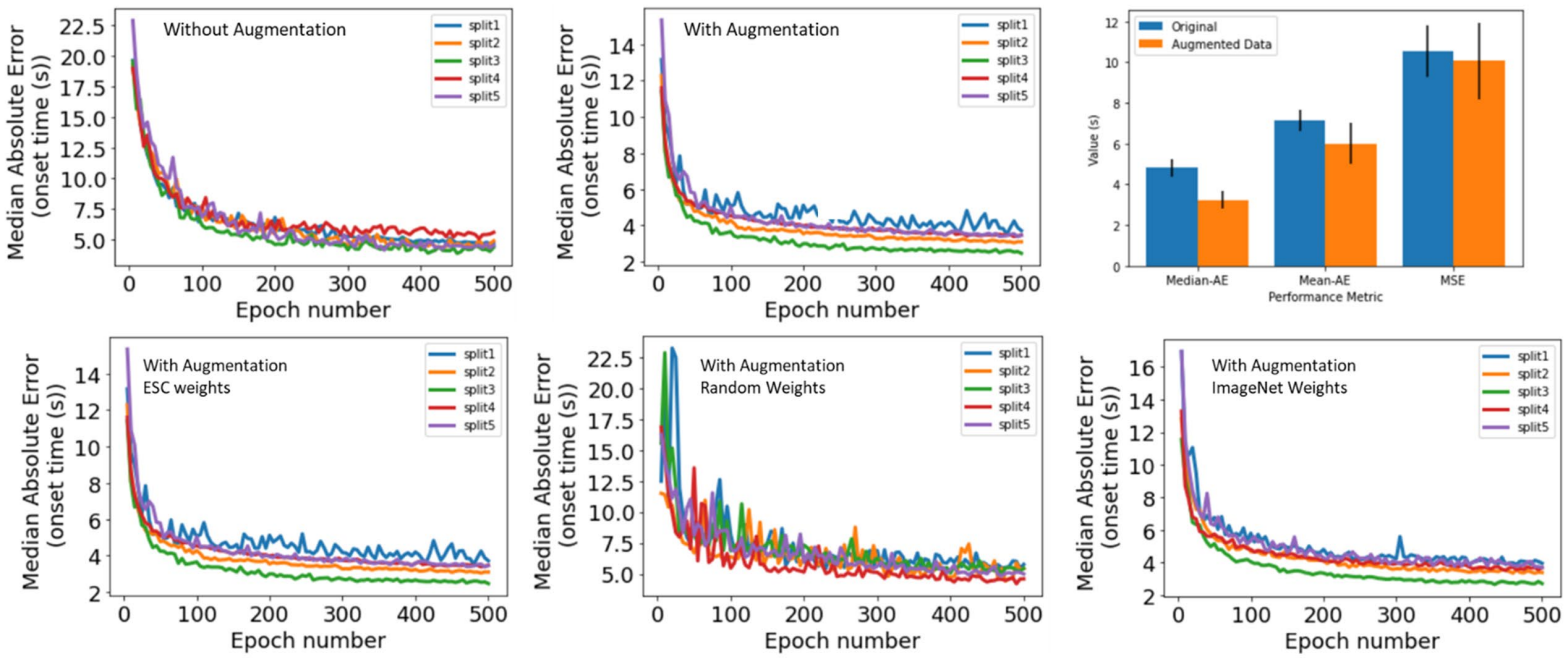
(Top) Seizure Onset Detection (SOD) model validation performance vs. training epochs on original (left) and augmented (center) datasets. Right panel shows median absolute error, mean absolute error, and root mean squared error. In all cases, better model performance was observed when model training was performed on the original + augmented datasets. (Bottom) Seizure Onset Detection model validation performance vs. training epochs observed by fine-tuning the electrographic seizure classifier (left), a ResNet50 model with random weight initialization (center), and a ResNet50 model with weight learned by training on the ImageNet dataset (right).

In addition to the median absolute error, mean absolute error and root mean squared error also improved when training was performed with the augmented dataset [Figure 6 (top), right panel].

#### 3.3.2. Comparing seizure onset detection performance with different initial model weights

**Figure 7:**
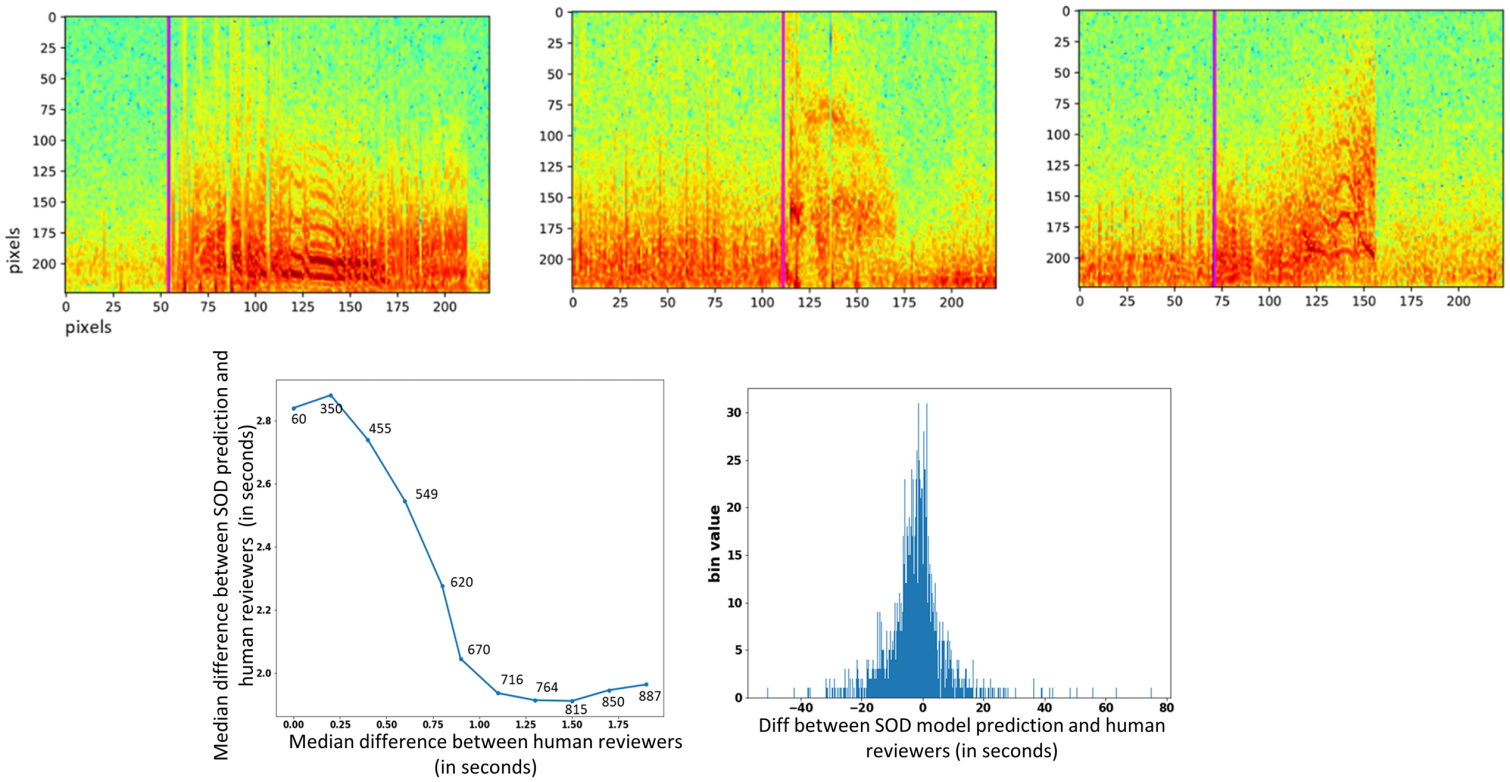
(Top) Seizure onset time prediction in 3 example iEEG channels. The pink vertical line shows the SOD model’s predicted onset time in each of the three spectrogram images. (Bottom) Left panel: median difference in onset times between SOD model and human reviewer (y-axis) vs. median difference in onset times between reviewers (x-axis). Numbers above data points show the number of iEEG channels used to compute the difference. Right panel: Histogram of difference between trained SOD model vs. human reviewer in seconds.

The Seizure Onset Detection (SOD) model was developed by fine-tuning the weights of the ESC model as described in Section 2. For comparison, a ResNet50 model with weights learned from training on the ImageNet dataset (Deng et al., 2009), and random weight initialization were also trained on the augmented datasets to assess if the ESC model initialization contributed to improvements in model performance. Validation errors from the three types of model initialization are shown in Figure 6 (bottom) and Table 6. As hypothesized, transfer-learning using the ResNet-50 model with initial weights from training on a relevant training task (ESC model) was significantly better (*p* < 0.05, paired *t*-test) compared to training with random weight initialization and ImageNet weight initialization. Specifically, an overall improvement in median absolute error of 1.6 s, and 0.4 s, respectively, was observed with ESC model weights compared to random and ImageNet weights. Figure 7 (top) shows three example seizure onset detections by a SOD model trained using the augmented dataset using ESC model weights as the starting point for training.

#### 3.3.3. Performance of the trained SOD prediction model on reviewer-annotated dataset

As described in Section 2, reviewers were asked to annotate seizure onset times on electrographic seizure iEEG channels. Table 7 shows the number of seizure onset times annotated by each of the three reviewers. A small percentage (8.3%, 8.5%, and 11.4%) of the electrographic seizures were not annotated with a valid onset time because the reviewers concluded that the electrographic seizure began before the start of the captured iEEG file.

**Table 7:** Number of seizure onset times annotated by the three reviewers.

	Reviewer 1	Reviewer 2	Reviewer 3
Number of iEEG channels with onset time annotated	1,615	1,570	2,081
Number of iEEG channels with onsets before iEEG record start (i.e., no valid onset time)	135 (8.36%)	133 (8.47%)	237 (11.39%)
Number of iEEG channels with onset times within iEEG record (i.e., with valid onset times)	1,516 (93.89%)	1,437 (91.53%)	1,844 (88.61%)
Number of iEEG channels with all three expert annotations within a 10-s window	887		
Number of iEEG channels with all three expert annotations within a 5-s window	670		
Number of iEEG channels with all three expert annotations within a 3-s window	549		

On iEEG channels where all three reviewers agreed that the onset time was within a 0.25 s window, the SOD model’s median absolute error was 2.9 s [Figure 7 (bottom)], and on iEEG channels when all three reviewers agreed that the onset time was within a 1.5 s window, the SOD model’s median absolute error was 1.7 s.

Across all iEEG records, the median absolute error between the SOD model and the reviewer annotations was 3.4 s. In comparison, a trivial model that uses the neurostimulator-detected episode start time as the seizure onset time, had a median absolute error of 8.3 s. Finally, a histogram of differences between SOD and reviewer scores [Figure 7 (bottom), right panel] shows that the model predictions were not biased in any one direction. That is, number of positive errors (or model prediction time > reviewer annotated onset time) and number of negative errors were comparable.

## 4. Discussion

This paper significantly adds to existing literature on leveraging deep learning and machine learning (AI) techniques for facilitating data review in epilepsy. First, it shows that AI models can be trained with the same level of iEEG seizure/non-seizure classification performance as board certified neurology and clinical neurophysiology and epilepsy experts. Second, it quantifies expert agreement levels with each other and shows that experts tend to agree more on iEEG records captured on leads implanted in the MTL regions compared to neocortical regions. Similarly, the trained electrographic seizure models also agreed more with reviewers on MTL channels compared to neocortical channels. Finally, it demonstrates how a second AI model can be trained on a related task of finding seizure onset times using a relatively small, labeled dataset by leveraging transfer learning (LeCun et al., 2015) of features learned by the first AI model, and through data augmentation techniques.

Overall, the Electrographic Seizure Classifier model’s agreement with the expert iEEG reviewers was on-par with experts’ agreement among themselves. This was particularly striking in the analysis of model prediction certainty vs. combined expert prediction certainty. On iEEG channels in which all three experts were in complete agreement, the model’s electrographic seizure and non-seizure prediction probabilities were also very high. For instance, on the 1,808 channels in which all three experts agreed on the non-seizure class, the ESC model agreed that the probability of a seizure on that channel was low, in the range of [0.12–0.19]. Similarly, on 1,417 channels in which all three experts agreed on the seizure class, the ESC model agreed that the probability of a seizure on that channel was high, in the range of [0.92–0.95]. When the experts were not in complete agreement, the ESC model’s probabilities reflected the disagreement among experts. For instance, for channels classified as a seizure by only one of three experts, the ESC model’s predicted seizure probability was in the range of [0.32–0.42], and when two of three experts classified channels as a seizure, the ESC model’s seizure probability was in the range of [0.61–0.70]. This shows that the trained model’s learned iEEG features and decision criteria for classifying iEEG records might be similar to those learned by human reviewers.

Just as human reviewers exhibit disagreement and uncertainty about seizure and non-seizure classifications, so does the ESC model, as reflected in its prediction probabilities. Hence, when building clinical applications using the Electrographic Seizure Classifier model (Barry et al., 2021), it may be useful to display model certainty scores, or seizure probability values, in addition to the predicted seizure or non-seizure class. This would allow a physician making use of the ESC model to manually review only those iEEG records where the model’s classification certainty is low and rely on the model’s classifications when the model’s confidence is high. Even though these results are very encouraging and demonstrate that the trained Electrographic Seizure Classifier’s performance matches that of trained experts, it should be noted that only three reviewers were recruited for labeling the ~1,000 test iEEG records. Resource and time constraints did not permit recruiting more experts or the review of a larger iEEG test dataset. Nevertheless, the clinical validation tests performed in this study demonstrate that the trained Electrographic Seizure Classifier model performs well enough to be practically useful in the clinical workflow. Continuous monitoring of the deployed model should be performed to ensure that the model performs well in different patient populations, and feedback received from users should be used to improve the model’s performance in the future.

The three reviewers’ had better agreement on seizure and non-seizure classifications on iEEG channels from MTL regions compared to neocortical regions. On average, expert iEEG classification agreement on MTL channels was 5.65% better than on neocortical channels. This difference may be partially due to homogeneity in MTL seizure patterns across patients, with seizure onsets frequently starting with either hypersynchronous spiking activity or low voltage fast activity. Neocortical seizures, on the other hand, tend to be more heterogeneous across patients (Spencer et al., 1992; Lee et al., 2000; Perucca et al., 2014). Similar to experts, the trained ESC models also had better classification performance on MTL vs. neocortical channels (95.48% vs. 88.46%). Overall, the ESC model’s classification performance was 7% better on MTL leads compared to neocortical leads. Training data for the ESC models consisted of 44% MTL patients, and 56% neocortical patients (Barry et al., 2021). Hence, there was no bias in ESC model training which could have possibly explained the performance differences in the two patient populations. One way to improve the model performance in neocortical patients could be to train a model specifically for the neocortical patient population, so that feature learning can be performed exclusively for this patient group.

Model operating point is an important parameter that needs to be selected based on the application of interest and can have a significant impact on the success of the clinical application (Jordan and Mitchell, 2015). In this paper, a few different values of model operating points were explored and, in general, a higher operating point of 0.8 led to a slightly better overall classification performance (93.19%) than the default value of 0.5 (92.52%). However, the two different operating points had a substantial impact on class-specific performances, with a higher operating point of 0.8 identifying 95.4% of non-seizure iEEG channels annotated by the experts, and a lower operating point of 0.5 identifying 95.2% of seizure iEEG channels. Hence, for problems requiring higher seizure class specificity (i.e., true positive rate), a higher operating point must be chosen, and a lower operating point chosen for problems requiring higher seizure class sensitivity (i.e., true negative rate).

No difference was seen between the ESC model’s classification performance on iEEG data captured with the two different models of the RNS Neurostimulator (RNS-300 M vs. RNS-320), which shows that the ESC model’s performance is robust across neurostimulator models. However, model performance monitoring tests should be performed periodically to ensure sustained performance levels on data captured with future new releases of the RNS System. If performance degrades in the future, it could suggest data drift and may require re-training the ESC models with iEEG data captured from the newer RNS System models.

Overall, iEEG record-level agreements between experts and the trained ESC models were similar to iEEG channel-level agreements (87.73% at a model operating point of 0.5, and 87.56% at a model operating point of 0.9). For an application to count the number of electrographic seizures, it may not be relevant to know which channel in the iEEG record contains seizures. Instead, it may be sufficient to classify the entire multi-channel iEEG record as seizure or non-seizure. For example, counts of electrographic seizure iEEG records captured after a change in epilepsy treatment (such as a change in an antiseizure medication) can be compared to counts before the change, to assess if the change had a favorable (or unfavorable) impact on the patient. Such a measure may be used in conjunction with other recently identified iEEG biomarkers (Skarpaas et al., 2018; Desai et al., 2019a; Khambhati et al., 2021; Sun et al., 2021) to efficiently supplement patient self-reports of clinical outcomes.

Manually annotating seizure onset times can be very time-consuming, even more so than annotating iEEG activity as seizure or non-seizure. This is because identifying the seizure onset times in iEEG channels requires zooming into the iEEG activity at multiple time and frequency scales, and thoughtful consideration of when seizure activity begins (Spencer et al., 1992; Lee et al., 2000; Perucca et al., 2014; Nune et al., 2019). In our previous study (Barry et al., 2021), an unsupervised clustering method was used for facilitating manual labeling of iEEG channels as seizure and non-seizure. With the clustering tool, 138,000 iEEGs records were annotated in roughly 320 h (Barry et al., 2021). A similar approach cannot be taken for labeling seizure onset times, since seizure onset times relative to the start of the iEEG record can vary substantially from one iEEG record to another, even within individual patients, and hence a clustering method would simply not work for seizure onset labeling. As an alternative, transfer learning and data augmentation techniques were explored for training a seizure onset detector with a relatively small, labeled dataset. Accordingly, seizure onset times were manually annotated in 4,859 seizure iEEG channels. Time-shifting of the signal within the iEEG channels resulted in a synthesized augmented dataset consisting of 30,768 iEEG channels. The RNS System is generally configured to store 90-s iEEG records with 60 s of pre-trigger activity and 30 s of post-trigger activity. Long episode iEEG records are usually triggered 30 s after a detection episode begins (i.e., long episode duration configured to 30 s). This contributes to a bias in the dataset for seizure onsets to occur around 30 s after the record start. To mitigate this bias and enlarge the dataset, time-shifting augmentation was performed. Consequently, Seizure Onset Detection (SOD) models trained on the original plus augmented dataset had significantly (*p* < 0.05 paired *t*-test) better performance (3.12s median absolute error or MAE) than models trained on only the original dataset (4.4 s MAE).

The utility of using a model pre-trained on a relevant task to improve SOD performance was explored (LeCun et al., 2015; Desai et al., 2019b). To test our hypothesis that using a model pre-trained on a relevant task could offer performance boosts, three types of model weight initializations for the same ResNet50 architecture were performed when training the SOD model. The first model was initialized using ESC model weights from the relevant task, the second used randomly initialized weights, and the third was initialized using weights learned by training on the ImageNet dataset (Deng et al., 2009), which consists of over 14 million training examples from 1,000 classes. Even though the ImageNet weights were derived by training the ResNet50 model on a much larger dataset, over 100 times larger than the ESC training set, model performance was significantly better (*p* < 0.05, paired *t*-test) when transfer-learning was performed with ESC weights as the starting point. This demonstrates the importance of using task-relevant weight initializations when fine-tuning models with small, annotated datasets. Future model development for iEEG datasets collected from the RNS System and other neuromodulation devices could use the ESC models as starting-points for transfer learning (available for download for academic use at: https://dabi.loni.usc.edu/dsi/000012) and avoid the resource-intensive task of annotating large iEEG datasets. As an example, the ESC model demonstrated a classification accuracy of 70% on scalp EEG data in the TUH EEG dataset, which is a large publicly available collection of scalp EEG recordings (Sun and Morrell, 2014). This accuracy level is higher than chance level (50%), indicating that the ESC model has learned features that can be applied to EEG datasets captured using different devices and recording techniques. By fine-tuning the ESC model with labeled EEG examples from these other devices, it is likely that the model’s performance on those datasets would significantly improve. Alternatively, the ESC model can also be used as a feature extractor without additional fine-tuning. For instance, it has been utilized as a feature extractor to identify patients with similar intracranial EEG (iEEG) seizures in the context of the RNS System (Nune et al., 2019).

The combined ESC and SOD models could be of great assistance in several clinical scenarios. The ESC model can identify the iEEG channels with electrographic seizures, and the SOD model can identify the seizure onset time. Since the goal of responsive stimulation is often to stimulate early in the electrographic seizure, information from the ESC and SOD models can potentially be combined to select the neurostimulator detection settings to best detect the iEEG patterns that indicate the seizure onset. Another potential scenario is to use these models to help physicians as they determine whether a patient might benefit from an epilepsy resection or ablation procedure (King-Stephens et al., 2015). The ESC model can be used to determine whether the seizures are unilateral or bilateral. If seizures are seen bilaterally, the SOD model can determine the hemisphere with the earliest seizure onset. If the patient consistently has seizures originating only from one hemisphere, or if the onset is consistently earlier in one hemisphere than the other, then the patient could be a candidate for an ablation or resection of that seizure onset zone (King-Stephens et al., 2015).

In summary, the work in this paper aims to clinically validate an Electrographic Seizure Classifier (ESC) model (Barry et al., 2021) and train a second Seizure Onset Detection (SOD) model using a relatively small, labeled dataset by leveraging features learned by the first model. There are abundant opportunities for improving the performance of both the ESC and SOD models described in this paper. For example, transformer-based models which have recently shown exceptional performance on tasks involving sequence data (Vaswani et al., 2017) may be a promising area to explore for iEEG data analysis. Nevertheless, clinical tools developed with the models presented in this paper could significantly improve epilepsy clinical workflows, particularly with the iEEG data review that is predominantly performed manually as of today. The ultimate goal is to provide physicians with information to assist them in their treatment of patients with challenging epilepsy.

## Data availability statement

The data presented in the study are deposited in Data Archive Brain Initiative (DABI) at https://dabi.loni.usc.edu/dsi/000012.

## Author contributions

SD led the study, performed analysis, and primarily wrote the paper. MA performed analysis presented in this study. WB annotated 4,859 iEEG channels used for training the seizure onset detection model. JK, SB, and CT annotated 1,000 iEEG records for clinical validation of the trained deep learning models. TT, CS, and MM helped with designing the experiments presented in this manuscript and provided feedback on the writing. MM was involved in selecting the validation data annotated by the three expert reviewers. All authors contributed to the article and approved the submitted version.

## Conflict of interest

SD, WB, TT, CS, MM are employees of NeuroPace. MA was an intern at NeuroPace when performing analysis presented in this manuscript. JK, SB, and CT were consultants at NeuroPace when they independently annotated 1,000 iEEG records.

## Publisher’s note

All claims expressed in this article are solely those of the authors and do not necessarily represent those of their affiliated organizations, or those of the publisher, the editors and the reviewers. Any product that may be evaluated in this article, or claim that may be made by its manufacturer, is not guaranteed or endorsed by the publisher.
