# Supplementary material for: Expert and deep learning model identification of iEEG seizures and seizure onset times
Source: Front Neurosci. 2023 Jul 5;17:1156838. doi: 10.3389/fnins.2023.1156838 (PMC10354337; doi:10.3389/fnins.2023.1156838)
Supplement: Supplementary file 1 [file Data_Sheet_1.DOCX]

Supplementary material

OP = model operating point

| All lead locations:  Training fold number (OP = 0.5) | Seizure agreement  (n = 1417) | Non-seizure agreement  (n = 1808) | Overall agreement |
| --- | --- | --- | --- |
| 1 | 95.48 | 92.26 | 93.87 |
| 2 | 96.19 | 86.56 | 91.37 |
| 3 | 94.35 | 87.78 | 91.07 |
| 4 | 95.48 | 91.26 | 93.37 |
| 5 | 94.5 | 91.32 | 92.91 |

| All lead locations:  Training fold number (OP = 0.8) | Seizure agreement  (n = 1417) | Non-seizure agreement  (n = 1808) | Overall agreement |
| --- | --- | --- | --- |
| 1 | 90.61 | 96.63 | 93.62 |
| 2 | 92.59 | 93.97 | 93.28 |
| 3 | 88.64 | 95.52 | 92.08 |
| 4 | 92.1 | 95.13 | 93.61 |
| 5 | 90.97 | 95.74 | 93.35 |

| MTL lead locations:  Training fold number (OP = 0.5) | Seizure agreement  (n = 626) | Non-seizure agreement  (n = 584) | Overall agreement |
| --- | --- | --- | --- |
| 1 | 96.33 | 96.75 | 96.54 |
| 2 | 97.76 | 90.92 | 94.34 |
| 3 | 96.49 | 94.01 | 95.25 |
| 4 | 97.6 | 93.84 | 95.72 |
| 5 | 97.12 | 94.01 | 95.57 |

| MTL lead locations:  Training fold number (OP = 0.8) | Seizure agreement  (n = 626) | Non-seizure agreement  (n = 584) | Overall agreement |
| --- | --- | --- | --- |
| 1 | 94.89 | 99.14 | 97.02 |
| 2 | 94.73 | 96.58 | 95.65 |
| 3 | 94.41 | 97.77 | 96.09 |
| 4 | 95.37 | 96.75 | 96.06 |
| 5 | 94.25 | 97.95 | 96.1 |

| NEO lead locations:  Training fold number (OP = 0.5) | Seizure agreement  (n = 338) | Non-seizure agreement  (n = 538) | Overall agreement |
| --- | --- | --- | --- |
| 1 | 92.9 | 86.43 | 89.67 |
| 2 | 93.2 | 80.67 | 88.92 |
| 3 | 90.83 | 82.34 | 86.59 |
| 4 | 89.94 | 87.17 | 88.56 |
| 5 | 89.94 | 87.17 | 88.56 |

| NEO lead locations:  Training fold number (OP = 0.8) | Seizure agreement  (n = 338) | Non-seizure agreement  (n = 538) | Overall agreement |
| --- | --- | --- | --- |
| 1 | 82.84 | 92.94 | 87.89 |
| 2 | 87.87 | 89.96 | 88.92 |
| 3 | 80.77 | 93.12 | 86.95 |
| 4 | 86.39 | 92.57 | 89.48 |
| 5 | 86.39 | 92.57 | 89.48 |

| MTL + NEO Lead locations:  Training fold number (OP = 0.5) | Seizure agreement  (n = 453) | Non-seizure agreement  (n = 686) | Overall agreement |
| --- | --- | --- | --- |
| 1 | 96.25 | 93.0 | 94.62 |
| 2 | 96.25 | 87.46 | 91.86 |
| 3 | 94.04 | 86.73 | 90.39 |
| 4 | 96.69 | 92.27 | 94.48 |
| 5 | 94.26 | 92.27 | 93.27 |

| MTL + NEO Lead locations:  Training fold number (OP = 0.5) | Seizure agreement  (n = 453) | Non-seizure agreement  (n = 686) | Overall agreement |
| --- | --- | --- | --- |
| 1 | 90.51 | 97.38 | 93.94 |
| 2 | 93.16 | 94.9 | 94.03 |
| 3 | 86.53 | 95.48 | 91.01 |
| 4 | 91.83 | 95.77 | 93.8 |
| 5 | 89.85 | 96.36 | 93.1 |
