# Supplementary material for: Expert and deep learning model identification of iEEG seizures and seizure onset times
Source: Front Neurosci. 2023 Jul 5;17:1156838. doi: 10.3389/fnins.2023.1156838 (PMC10354337; doi:10.3389/fnins.2023.1156838)
Supplement: Supplementary file 2 [file Data_Sheet_2.DOCX]

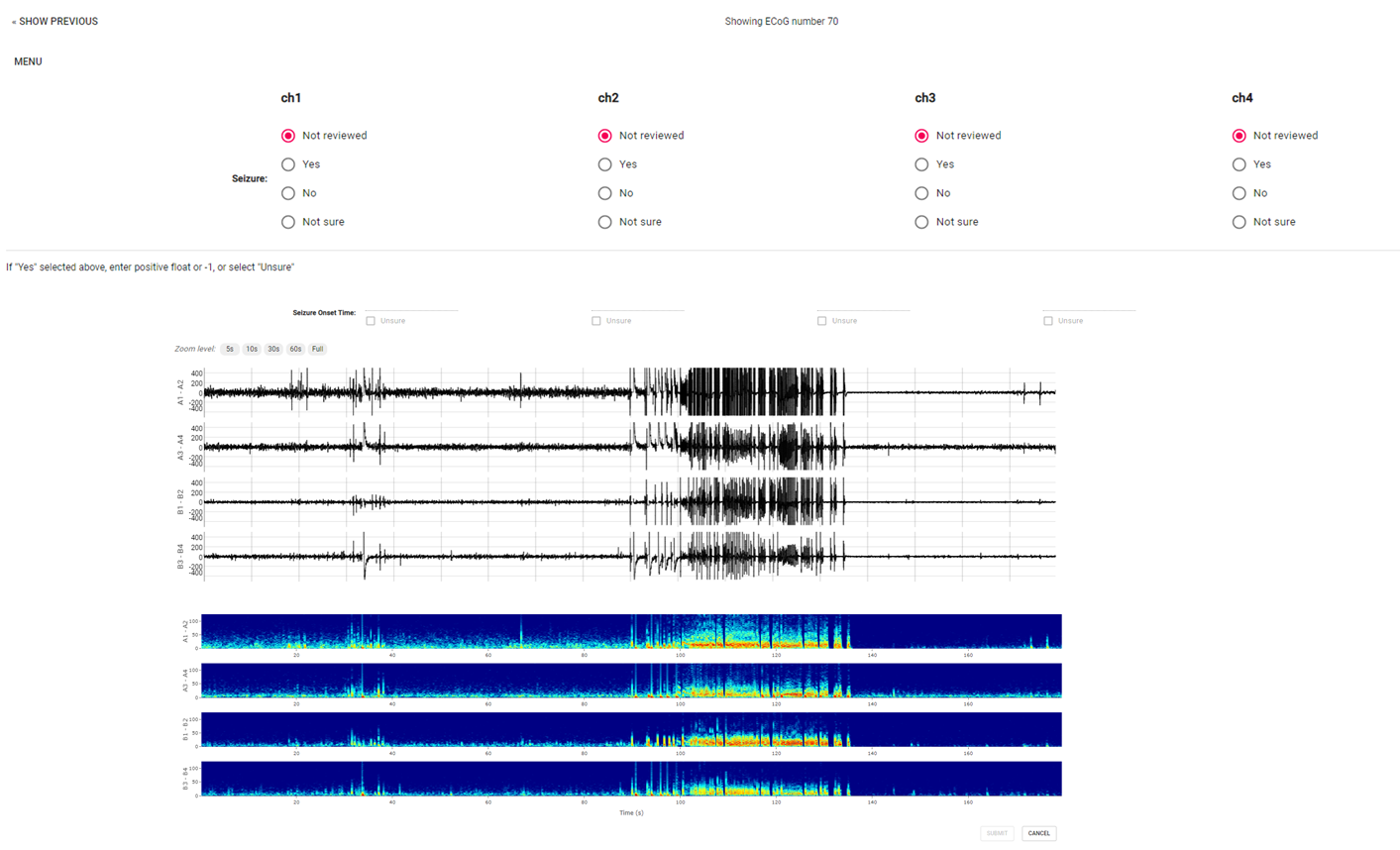


*Supplementary Figure 1. Screenshot of iEEG annotation tool showing time-series and spectrogram representation of a 4-channel iEEG record. The tool was loaded with 1,000 iEEG records from 100 patients and presented to each of the three reviewers who independently labeled the iEEG channels. When reviewers labeled an iEEG channel as electrographic seizure, the tool also requested a seizure onset time annotation for that channel.*


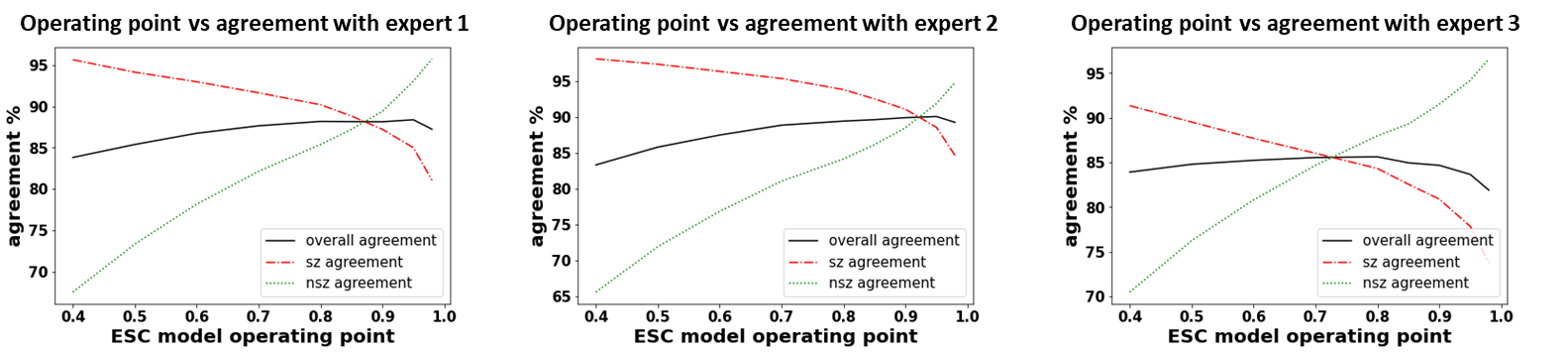


*Supplementary Figure 2. iEEG record classification performance of the ESC model against each of the three reviewers at varying model operating points.*
